# Inkjet Printing of Carbon Nanotubes

**DOI:** 10.3390/nano3030453

**Published:** 2013-07-29

**Authors:** Ryan P. Tortorich, Jin-Woo Choi

**Affiliations:** 1 School of Electrical Engineering and Computer Science, Louisiana State University, Baton Rouge, LA 70803, USA; E-Mail: rtorto1@tigers.lsu.edu; 2 Center for Advanced Microstructures and Devices, Louisiana State University, Baton Rouge, LA 70803, USA

**Keywords:** carbon nanotube ink, carbon nanotube patterning, inkjet printing, flexible electronics

## Abstract

In an attempt to give a brief introduction to carbon nanotube inkjet printing, this review paper discusses the issues that come along with preparing and printing carbon nanotube ink. Carbon nanotube inkjet printing is relatively new, but it has great potential for broad applications in flexible and printable electronics, transparent electrodes, electronic sensors, and so on due to its low cost and the extraordinary properties of carbon nanotubes. In addition to the formulation of carbon nanotube ink and its printing technologies, recent progress and achievements of carbon nanotube inkjet printing are reviewed in detail with brief discussion on the future outlook of the technology.

## 1. Introduction

Carbon nanotubes (CNTs) have truly become one of the most exciting materials in recent years due to their extraordinary properties. In particular, the electrical properties of carbon nanotubes lend themselves to many applications including use in transistors [1,2], radio-frequency identification (RFID) tags [3], sensors [4,5,6,7,8], photonics [9,10], biological sensing labels [11], and more. One of the most interesting applications of carbon nanotubes is that of transparent electrodes. Considering indium tin oxide (ITO) is the dominant commercial material for transparent electrodes, carbon nanotubes would provide a cheaper alternative. Furthermore, not only can carbon nanotubes assist in reducing the cost of these types of electronic devices, but they can also allow these devices to become flexible.

In order to take advantage of the unique properties of carbon nanotubes, many groups have experimented with various carbon nanotube deposition methods such as dip coating [12], spray coating [2,8,13,14], electrophoretic deposition [15], and others. However, one of the prominent methods of interest today is carbon nanotube printing. There have been demonstrations of screen printing [16], aerosol printing [17,18,19], transfer printing [20], and contact printing [21] to deposit carbon nanotubes on various substrates, but the most favorable form of printing is that of inkjet printing.

Inkjet printing offers unique advantages over other methods of printing. It requires absolutely no prefabrication of templates, allowing for a rapid printing process at low cost. Additionally, due to its precise method of patterning, post-printing steps are not necessary. Furthermore, multiple materials can be deposited simultaneously with the use of multiple ink cartridges, and the amount of deposited material can be controlled with great precision. Finally, due to the nature of inkjet printing technology, multiple layers can be printed on top of one another with great ease. Inkjet printing is currently being used to deposit various types of conductive nanomaterials such as gold [22,23] and silver [24,25]. Although these metals are excellent conductors, carbon nanotubes are cheaper and more versatile in the sense that they can behave as both a semiconductor and a conductor.

Before discussing inkjet printing as it pertains to carbon nanotube printing, it is first necessary to review the various inkjet printing technologies. In general, inkjet printing can be split into two categories, namely continuous and drop-on-demand. As suggested by its name, continuous inkjet printing supplies a continuous stream of ink droplets. These droplets are charged upon leaving the nozzle and are then deflected by voltage plates, where the applied voltage determines whether the droplet will be deposited onto the substrate or recycled through the gutter. Consequently, when the printer is not actually printing anything onto a substrate, a stream of droplets is still being ejected from the nozzle and recycled through the gutter.

While continuous inkjet printers are still used, drop-on-demand inkjet printers are more common. As opposed to a continuous inkjet printer, a drop-on-demand inkjet printer ejects a droplet of ink only when it is told to do so. Therefore, when the printer is not actually printing anything onto a substrate, there are no droplets being ejected from the nozzle. Drop-on-demand inkjet printers can be further split into two categories, namely thermal and piezoelectric. Thermal inkjet printers, sometimes referred to bubble jet printers, contain a thin film resistor in the nozzle. In order to eject a droplet, this thin film resistor is heated by passing current through it. This causes the ink in the nozzle to vaporize, creating a bubble and a large increase in pressure, which forces ink droplets out of the nozzle. Hewlett-Packard, Canon, and Lexmark employ this type of drop-on-demand inkjet printer.

Piezoelectric inkjet printers contain a piezoelectric transducer in the nozzle. When voltage is applied to the piezoelectric transducer, it deforms and causes an increase in pressure, which forces ink droplets out of the nozzle. In terms of consumer printers, Epson employs this type of drop-on-demand inkjet printer. However, many specialized commercial inkjet printers, such as the Fujifilm Dimatix, employ the piezoelectric drop-on-demand technology as well.

Although inkjet printing has its advantages, it also has its obstacles and difficulties. The first step in inkjet printing is formulating ink. There are several issues to consider when mixing ink to be used in an inkjet printer. In general, the ink must maintain a low surface tension as well as a low viscosity. Aside from these properties, incorporating nanomaterials into an ink presents further issues, primarily due to the difficulty of dispersing the nanomaterial within the ink. More specifically, a well-dispersed nanomaterial ink should be free from flocculation of the nanomaterial within the ink. There is a great deal of current research being done on carbon nanotube dispersion, and there have been reports on dispersing carbon nanotubes in water [26,27,28,29,30,31,32,33] as well as organic solvents such as dimethylformamide (DMF) [26,34,35], N-methyl-2-pyrrolidone (NMP) [26,35,36], chloroform [26,37], and others [35].

With the basics of inkjet printing covered, the specifics of carbon nanotube inkjet printing will now be discussed in the following sections. This includes carbon nanotube networks, formulation and preparation of carbon nanotube ink, and key aspects of ongoing research. Along the way, advantages and disadvantages will be discussed for varying methods.

## 2. Carbon Nanotube Network

**Figure 1:**
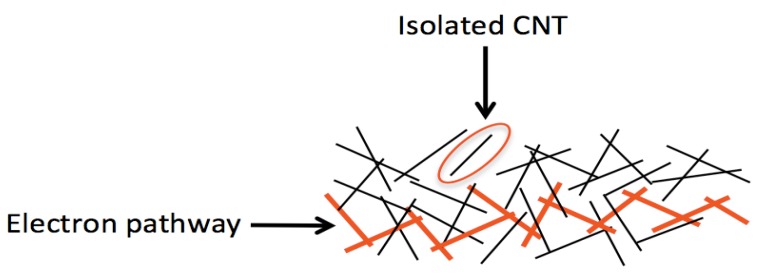
Random carbon nanotube network showing both isolated carbon nanotube and formation of electron pathway via overlapping carbon nanotubes.

Before reviewing both the difficulties in formulating carbon nanotube ink and the current research, it is important to first understand how inkjet printing of carbon nanotubes can be used to create conductive traces. When carbon nanotubes, or any one-dimensional nanomaterial, are printed onto a substrate, the solvent evaporates, leaving behind a random network of carbon nanotubes. This network would be analogous to dropping a handful of spaghetti onto a tabletop. Some of the spaghetti might not be in contact with any other spaghetti. In a similar way, some of the carbon nanotubes might be completely isolated without having contact with any other carbon nanotubes. In this case, electrons are confined to a single carbon nanotube. Consequently, isolated carbon nanotubes do not contribute to the conductivity of the printed film. On the other hand, some of the spaghetti may indeed be in contact with other spaghetti, just as some of the carbon nanotubes may be in contact with other carbon nanotubes. This essentially creates an electron pathway. Electrons are capable of traveling from one carbon nanotube to another, ultimately resulting in current, which is the reason for the conductivity of the printed film. Figure 1 demonstrates this concept.

**Figure 2:**
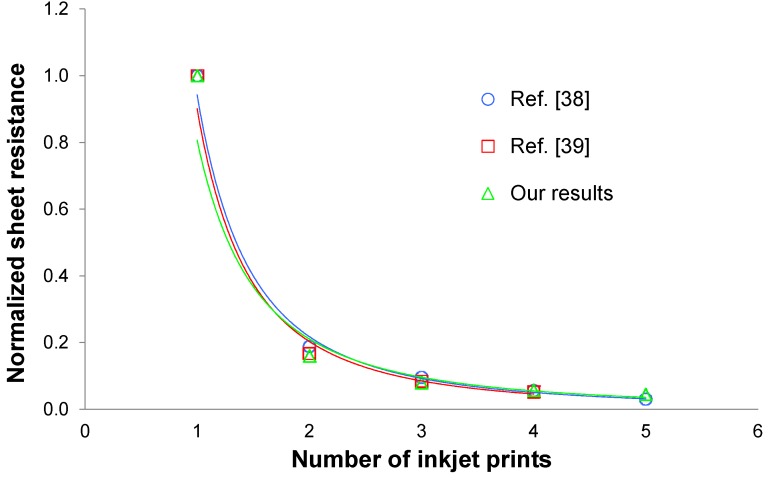
Estimated sheet resistance versus number of prints for two recent reports on carbon nanotube inkjet printing [38,39] and our own recent test results on carbon nanotube inkjet printing. Sheet resistance values are normalized.

As expected, the amount of current is directly related to the number of electron pathways. This suggests that the length of carbon nanotubes plays an important role in the conductivity of a carbon nanotube thin film. Revisiting the aforementioned pasta analogy, if the spaghetti pieces are short, the probability of them touching each other decreases. For a carbon nanotube network, this corresponds to a lower conductivity. On the contrary, if the spaghetti pieces are long, the probability of them touching increases, which corresponds to a higher conductivity in a carbon nanotube network. It will soon be shown that in order to achieve highly conductive films of carbon nanotubes by inkjet printing, it is necessary to print multiple layers of carbon nanotubes. This initially results in a substantial increase in conductivity since each additional layer of carbon nanotubes provides a denser network and produces more electron pathways. However, eventually, the conductivity of the printed film will reach the carbon nanotube bulk conductivity. Figure 2 shows estimated data from two recent reports on carbon nanotube inkjet printing [38,39] as well as our own recent test results. It should be noted that sheet resistance is plotted, which is both more common and useful than conductivity.

The bulk conductivity is determined by many factors. Here again, the length of the carbon nanotubes also plays a major role. As demonstrated by Hecht *et al*., the conductivity of a carbon nanotube network increases as the length of the carbon nanotubes increases [40]. Additionally, the type of carbon nanotubes used affects the conductivity as well, which includes single-walled or multi-walled, semiconducting or metallic, pristine or functionalized, and other variations. Further, dispersants can even reduce the conductivity of carbon nanotube networks by inhibiting contact between carbon nanotubes [41]. Finally, the drying process can affect the distribution and orientation of carbon nanotubes, which will be discussed in Section 4.4. Nevertheless, this random network of carbon nanotubes is essential in forming a conductive thin film.

## 3. Carbon Nanotube Ink

There are many issues that need to be taken into account regarding carbon nanotube ink. First and foremost, carbon nanotube dispersion is a major obstacle. Aside from dispersion, surface tension and viscosity are important characteristics for carbon nanotube ink. Finally, preparation of carbon nanotube ink involves multiple steps to ensure well-dispersed carbon nanotubes and removal of any carbon nanotube bundles.

### 3.1. Carbon Nanotube Dispersion

Due to the nature of the material, carbon nanotubes are quite difficult to disperse in a liquid. The van der Waals forces between carbon nanotubes can easily cause agglomeration and sedimentation, which is highly undesirable due to the possibility of clogging the inkjet nozzle. As a result, many groups have experimented with various methods of carbon nanotube dispersion through the use of sidewall functionalization, organic solvents, and dispersants in the case of water-based ink.

#### 3.1.1. Functionalized Carbon Nanotube Dispersion

The first method for carbon nanotube dispersion is that of sidewall functionalization. This entails a chemical process whereby molecules are bound to the carbon nanotube sidewalls. One of the most common methods of carbon nanotube functionalization used to enhance dispersion is called carboxylation. In carboxylation, carboxyl groups (–COOH) are attached to the sidewalls of carbon nanotubes through a series of chemical steps. Unlike the hydrophobic carbon nanotube sidewalls, these carboxyl groups are hydrophilic, reducing the possibility for carbon nanotube bundling. On the downside, the chemical steps necessary for functionalization tend to introduce defects into the carbon nanotube sidewalls. This effectively decreases the conductivity of the carbon nanotubes. Nevertheless, a few groups have successfully formulated and printed carbon nanotube ink using functionalized carbon nanotubes [41,42,43].

#### 3.1.2. Organic Solvent-Based Carbon Nanotube Dispersion

In order to avoid hindering the conductive nature of carbon nanotubes, other means of dispersion can be used. For example, organic solvents are superb in their ability to disperse carbon nanotubes. There is no need for functionalization of the carbon nanotube sidewalls or addition of other materials to enhance dispersion. Rather, the solvent itself works as a dispersant. The organic solvent molecules adsorb onto the carbon nanotube surface due to a hydrophobic interaction, countering the strong van der Waals forces between the nanotubes [34]. Additionally, due to their inherent low surface tension, there is no need to add a wetting agent to organic solvent-based inks. On the contrary, many organic solvents present some issues for practical use. First, it has been reported that organic solvents have a carbon nanotube concentration limit of approximately 0.1 mg/mL [44]. Organic solvents also tend to be quite volatile, which can cause problems both when the ink is being prepared and when the ink is being used in a cartridge. Unless the cartridge is sealed properly, the solvent will evaporate, leaving behind nothing but the carbon nanotubes and ultimately clogging the nozzle. Another problem encountered when dealing with organic solvents is that of health and environmental effects. If the proper precautions are not taken, there may be some serious consequences. Lastly, organic solvents can be very corrosive to certain polymer materials. As a result, cartridges used for organic solvent-based carbon nanotube ink must be made of materials that resist their corrosive property. This corrosive characteristic also limits the substrate selection for organic solvent-based carbon nanotube inks. Despite the difficulties, many groups have successfully developed and printed carbon nanotube inks using organic solvents such as DMF [3,38,45,46,47,48] and NMP [49].

#### 3.1.3. Water-Based Carbon Nanotube Dispersion

In addition to organic solvent-based carbon nanotube inks, some groups have developed and printed water-based carbon nanotube inks with the use of dispersants rather than functionalization of the carbon nanotubes [39,50,51,52,53,54]. These water-based inks are environmentally friendly, easy to store, and safer to handle. However, water-based inks are much more difficult to develop since carbon nanotubes do not readily disperse in water without the aid of additional dispersants. As the surface of carbon nanotubes is hydrophobic, the nanotubes do not want to be in contact with water. Rather, they bundle together due to the attractive van der Waals forces.

**Figure 3:**
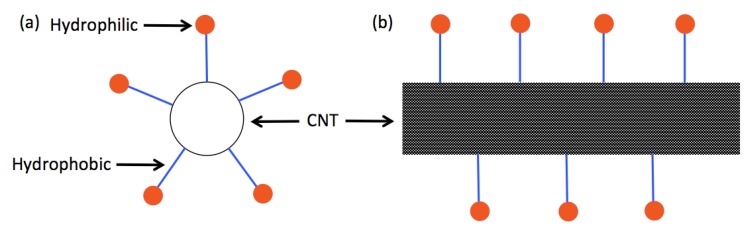
Surfactant-assisted dispersion of carbon nanotubes: (**a**) cross-section of carbon nanotube; and (**b**) side view of carbon nanotube.

There are a few ways to overcome these strong van der Waals forces. Aside from sidewall functionalization, surfactants and polymers can be used to cover the surface of each carbon nanotube in order to negate the strong van der Waals forces. This is achieved through both physical and chemical means. Surfactants are amphiphilic molecules having a hydrophilic head and a hydrophobic tail. Thus, when a surfactant is introduced into a water-based carbon nanotube ink, the surfactant molecules adsorb onto the surface of each carbon nanotube due to the hydrophobic tail. This essentially forms a barrier around the perimeter of the carbon nanotube, which acts as the physical means to negate the van der Waals forces when carbon nanotubes are in close proximity to each other. Additionally, because the outer layer of the surfactant-covered carbon nanotube consists of the hydrophilic heads, there is a repulsive chemical force between each carbon nanotube. Polymers, on the other hand, are long chains of monomers that wrap around the carbon nanotubes forming a helix. In a similar fashion to surfactants, polymers provide both a physical and a chemical means for overcoming the van der Waals forces. Figure 3 briefly illustrates how surfactants adsorb onto the carbon nanotube surface.

### 3.2. Carbon Nanotube Ink Surface Tension

In order for an ink droplet to be ejected from the nozzle, the ink must maintain a low surface tension. Due to the extremely small volume of ink being ejected from the nozzle (in the pl range), a low surface tension is absolutely necessary. If the surface tension is too high, the ink droplets may remain in the nozzle of the cartridge, which is highly undesirable.

**Figure 4:**
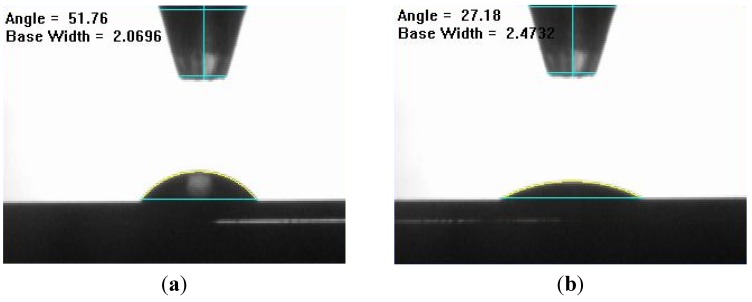
Effect of surfactant on surface tension: (**a**) 3 µL droplet of water without surfactant; and (**b**) 3 µL droplet of water with surfactant. Surfactant clearly decreases the surface tension of the droplet.

As mentioned in Section 3.1.2, organic solvents already have a low surface tension, so they do not require the addition of wetting agents. However, unlike organic solvents, water has a very high surface tension, resulting from the strong cohesive interaction between water molecules. In order to combat this high surface tension, wetting agents are used to lower the surface tension. Typically, surfactants are used as the wetting agent in carbon nanotube inks. In a liquid like water, the surfactant molecules accumulate on the water-air interface due to their amphiphilic structure. This ultimately reduces the cohesive forces between water molecules at the surface, which results in lower surface tension, allowing the water to spread out more on a given surface as shown in Figure 4.

### 3.3. Carbon Nanotube Ink Preparation

After determining the ingredients and relative concentrations for the carbon nanotube ink, a series of steps are performed in order to obtain useable ink. First, the ink needs to be mixed in order to disperse the carbon nanotubes within the liquid. This can be done in many ways, but the most common approach for dispersing carbon nanotubes is sonication, which uses high frequency vibrations to separate carbon nanotubes within a liquid. Although it works very well, this method also has its drawbacks. In particular, sonication can both shorten carbon nanotubes and cause defects. In the former case, shorter carbon nanotubes reduce the probability of forming an electron pathway in a carbon nanotube network, which can decrease the conductivity of the printed film. In the latter case, defects can negatively affect the inherent conductivity of the carbon nanotubes. Finally, sonication can both physically and chemically affect the solvent and dispersants used in a carbon nanotube ink [44]. Nevertheless, sonication seems to be the primary method of choice for carbon nanotube dispersion.

After dispersing the carbon nanotubes, the ink is centrifuged in order to separate the well-dispersed carbon nanotubes from the bundles or agglomerations, which could clog the printer nozzle. The supernatant solution is then collected and may be centrifuged again. Sometimes, the carbon nanotube ink is also filtered in order to further remove any bundles of carbon nanotubes that could clog the printer nozzle. The filtering step may be performed multiple times to ensure a uniform and well-dispersed carbon nanotube ink. Once the ink is formulated, it is loaded into an inkjet cartridge and ready to be printed.

## 4. Carbon Nanotube Inkjet Printing

One of the earlier demonstrations of carbon nanotube inkjet printing was reported by Fan *et al*. in 2005 [39], but one of the more recognized works was reported by Kordás *et al*. in 2006 [43]. Since then, there have been numerous displays of carbon nanotube inkjet printing, all of which have been successful in producing conductive carbon nanotube films. Rather than providing an exhausting review of each and every demonstration, a few key aspects of current research are discussed in this section.

### 4.1. Inkjet Printers

As stated previously, there are multiple types of inkjet printers, and all of them have been used for carbon nanotube printing. Consumer inkjet printers are quite cheap and offer familiarity, so there is no need to learn new software or hardware. Nevertheless, these printers are made to print a specific type of ink, so developing useable ink can be a bit more difficult. The new ink must match the original ink in all aspects. Furthermore, in the instance where a new ink clogs the nozzle, some consumer inkjet printers are easier to clean than others. In general, each Hewlett-Packard printer cartridge has its own nozzle, allowing the user to easily remove the cartridge and clean it. On the other hand, the nozzle for Epson printer cartridges is built into the printer itself and cannot be easily removed for cleaning. The most prominent disadvantage for consumer inkjet printers is their overall lack of control. In particular, the drop volume and spacing cannot be adjusted, and the resolution is relatively low. Regardless of these issues, there have been successful demonstrations of printing carbon nanotubes with consumer inkjet printers.

Commercial inkjet printers like the popular Fujifilm Dimatix are specifically made for printing various types of materials. As a result, they have a great deal of control over drop volume and spacing, and they provide better resolution. Although these specialized inkjet printers can be expensive, they seem to be a good choice for carbon nanotube printing due to their superior functionality.

### 4.2. Sheet Resistance

In order for carbon nanotube films to replace other metallic conductors, they must maintain a comparable sheet resistance. Some groups were able to achieve a sheet resistance below 1 kΩ/□ using multiple layers of carbon nanotubes, the lowest being 78 Ω/□ with a total of 200 prints demonstrated by Chen *et al*. [53]. Although this is a very low sheet resistance, performing 200 prints is not ideal. Taking the print number into account, the lowest recorded sheet resistance is 760 Ω/□ with a total of 12 prints [50].

One key factor that can play a major role in sheet resistance is that of dispersants. As mentioned in Section 2, dispersants can reduce the conductivity of carbon nanotube thin films. When dispersants are used in carbon nanotube ink, they form a physical barrier around the carbon nanotubes. In a carbon nanotube network, these dispersants can inhibit the contact between carbon nanotubes, possibly resulting in a very large decrease in conductivity. In order to diminish this effect, the dispersant concentration can be decreased. Consequently, there may be a reduction in the amount of dispersant that covers each nanotube, allowing for better contact between carbon nanotubes. On the contrary, decreasing the dispersant concentration can also result in a lower concentration of carbon nanotubes, which subsequently decreases the conductivity. Another possible way to prevent dispersants from reducing the conductivity is by simply removing them. Many dispersants are soluble in water and other liquids. By placing the substrate into one of these solvents, the dispersants may detach from the carbon nanotubes and dissolve into the liquid. It should be noted that during this process, some carbon nanotubes might detach from the substrate as well and disperse in the solvent, which can significantly reduce the conductivity.

### 4.3. Transparency

Aside from sheet resistance, in order for carbon nanotube films to replace transparent electrodes like indium tin oxide, they must maintain a comparable transparency. This involves a delicate balance because increasing the conductivity through multiple prints directly affects the transparency. As more and more carbon nanotubes are deposited onto a given substrate, the film becomes less and less transparent. Although Chen *et al*. were able to achieve a very low sheet resistance, the transmittance was only 10% [53]. To the authors’ knowledge, there has not been a report of carbon nanotube inkjet printing that demonstrates both good sheet resistance and good transparency. However, Mustonen *et al*. did accomplish this task using a composite ink made of carboxyl functionalized single-walled carbon nanotubes (SWCNT-COOHs) and poly(3,4-ethylenedioxythiophene)-poly(styrenesulfonate) (PEDOT-PSS) [55]. With 25 prints, the conductive film reached a sheet resistance of 1 kΩ/□ and a transmittance of 70%.

### 4.4. The Coffee Stain Effect

The well-known phenomenon denoted as the coffee stain effect occurs as a droplet of ink dries on the substrate. During drying, carbon nanotubes are pushed to the perimeter of the droplet due to an internal flux [56]. Denneulin *et al*. even demonstrated that carbon nanotubes orient themselves in specific directions at the perimeter of a drying ink droplet [57]. In order to overcome this, Denneulin *et al*. used a SWCNT-COOH/PEDOT-PSS composite ink. Other methods for limiting the coffee stain effect include heating the substrate and treating the substrate surface, which can both accelerate the drying process [38]. This helps to prevent flocculation of carbon nanotubes, allowing for a more uniform distribution and ultimately a more conductive film.

Seeing that carbon nanotube inkjet printing is quite involved, Table 1 on the following page provides a side-by-side comparison of recent reports on carbon nanotube inkjet printing.

**Table 1:** Comprehensive comparison of recently reported carbon nanotube printing.

Reference Number	Cited Papers	Printer	Solvent	Dispersant and Concentration	CNT Type and Concentration	Preparation	Best Sheet Resistance	Notable Feature
**[43]**	[39]	Canon	Water	Functionalized	MWCNT-COOH 0.26 mg/mL	Sonication Stirring Centrifuge	40 kΩ/□ 90 prints	One of the first reported
**[42]**	[43,55]	Dimatix	Water	Functionalized	SWCNT-COOH 0.1 mg/mL	Sonication Centrifuge	Not reported	FET-like behavior
**[41]**	[42,43,49]	Dimatix	Water	Functionalized	SWCNT-COOH (carboxylic acid) SWCNT-CONH_2_ (amide) SWCNT-PEG (polyethylene glycol) SWCNT-PABS (polyaminobenzene sulfonic acid) 0.13 mg/mL	Sonication Centrifuge	Estimated 2 kΩ/□ (for COOH and PABS) 14 prints (Assumed)	Fully inkjet printed FET
**[46]**	-	MicroJet	DMF	n/a	SWCNT 20 µg/mL	Centrifuge	Not reported	Gas sensing
**[47]**	-	MicroJet	DMF	n/a	SWCNT 0.01 mg/mL	Sonication	Not reported	Field emission display
**[3]**	[38,46]	Dimatix	DMF	n/a	SWCNT 0.4 mg/mL	Sonication	Estimated 150 Ω/□ 25 prints	RFID and gas detection
**[38]**	[43]	MicroJet	DMF	n/a	SWCNT 0.02 mg/mL	Centrifuge	Estimated 333 Ω/□ 8 prints	Uniform CNT network
**[45]**	[48,58]	MicroJet	DMF	n/a	SWCNT 0.001 µg/mL or 0.04 μg/mL (Assumed)	Sonication Filtering	Not reported	Doping of CNT Films
**[48]**	[42,43,49]	MicroJet	DMF	n/a	SWCNT 0.001 µg/mL or 0.04 μg/mL	Sonication Centrifuge Filtering	Not reported	Fully inkjet printed FET
**[49]**	[43]	Microdrop Autodrop	NMP	n/a	SWCNT 0.003 mg/mL	Sonication Centrifuge Filtering	Not reported	Use of CNT as active layer in TFT
**[39]**	-	Not reported	Water	Special dispersant S27000	MWCNT 3 mg/mL	Centrifuge Sonication	11.6 k Ω/□ 4 prints	One of the first reported
**[52]**	-	HP	Water	Gellan gum or xanthan gum <1 mg/mL	SWCNT or MWCNT Concentration not reported	Sonication	Not reported	Water vapor detection
**[53]**	[38,43,59]	Epson	Water	SDS 10 mg/mL	SWCNT 0.2 mg/mL	Sonication Centrifuge	78 Ω/□ 200 prints	Supercapacitors
**[50]**	[39,41,43,51,57,60]	Epson	Water	Combination of 3 different dispersants 150 mg/mL	MWCNT 0.15 mg/mL	Mixing Ball-milling Centrifuge	760 Ω/□ 12 prints	Low sheet resistance
**[51]**	[38,39,43,47,49,52,59]	Microfab	Water	Solsperce® 46000 5 mg/mL Byk 348 1 mg/mL	MWCNT 10 mg/mL	Sonication	Not reported	Electroluminescent device

Table 1 includes information such as the printer used, the ink ingredients and preparation, and the sheet resistance if reported. For consistency, concentrations that were reported as a weight percent were converted to a mass per volume value (*i.e.*, μg/mL or mg/mL). Also, the table has been organized into three sections based on how the carbon nanotubes were dispersed, namely functionalization, organic solvent, or water with a dispersant.

## 5. Conclusions and Future Outlook

Although carbon nanotube inkjet printing is relatively new, it seems to be a very promising method for deposition. Of course, there are a few obstacles to overcome before inkjet printing will become a commercial method for depositing carbon nanotubes, but it will not take long. With ongoing research in the area of carbon nanotube dispersion, stable carbon nanotube inks will soon be available. Furthermore, commercial inkjet printers like the Fujifilm Dimatix offer better control and resolution than general office inkjet printers. Takagi *et al.* have even demonstrated a method for further enhancing inkjet printing resolution by substrate surface modification [61]. In terms of applications, carbon nanotube inkjet printing can be used to fabricate transistors [41,48,49], sensors [3,46], electroluminescent devices [51], and more. Also, given the current progress, carbon nanotubes seem to be a potential candidate for next generation printable, flexible, and transparent electrodes.
